# First evidence of microbial wood degradation in the coastal waters of the Antarctic

**DOI:** 10.1038/s41598-020-68613-y

**Published:** 2020-07-29

**Authors:** Charlotte G. Björdal, Paul K. Dayton

**Affiliations:** 1 Dept. of Marine Sciences, University of Gothenburg, Carl Skottbergs Gata 22B, 405 30 Gothenburg, Sweden; 2 Scripps Institution of Oceanography, 9500 Gilman Drive, Mail Code 0227, La Jolla, CA 92093 USA

**Keywords:** Fungi, Bacteria, Marine microbiology, Ocean sciences

## Abstract

Wood submerged in saline and oxygenated marine waters worldwide is efficiently degraded by crustaceans and molluscs. Nevertheless, in the cold coastal waters of the Antarctic, these degraders seem to be absent and no evidence of other wood-degrading organisms has been reported so far. Here we examine long-term exposed anthropogenic wood material (Douglas Fir) collected at the seafloor close to McMurdo station, Antarctica. We used light and scanning electron microscopy and demonstrate that two types of specialized lignocellulolytic microbes—soft rot fungi and tunnelling bacteria—are active and degrade wood in this extreme environment. Fungal decay dominates and hyphae penetrate the outer 2–4 mm of the wood surface. Decay rates observed are about two orders of magnitude lower than normal. The fungi and bacteria, as well as their respective cavities and tunnels, are slightly smaller than normal, which might represent an adaptation to the extreme cold environment. Our results establish that there is ongoing wood degradation also in the Antarctic, albeit at a vastly reduced rate compared to warmer environments. Historical shipwrecks resting on the seafloor are most likely still in good condition, although surface details such as wood carvings, tool marks, and paint slowly disintegrate due to microbial decay.

## Introduction

Wood is mainly composed of cellulose, lignin and hemicellulose. These organic components are arranged in complex macro- and micro-fibrils forming the cell wall matrix of each wood fibers. The high concentration of lignin (around 25%) makes wood a very difficult substrate to degrade and only a few specialized organisms in nature have this capacity^1^. In marine waters wood degrading organisms can be divided into two groups. The first group consist of macro-organisms (mainly bivalves and crustaceans) that physically gnaw and feed on wood material. The second group are micro-organisms, fungi and bacteria, which by enzymatic processes dissolve and utilize the carbohydrates within the wood cell wall^2^.

Shipworms (Teredinidae and Xylaphagainae) belong to the group of bivalves known as the most aggressive degraders of wood in oxygenated and saline waters world-wide. By forming large internal tunnels they are able to transform solid wooden boards to a perforated material within a few years. This results in a rapid physical breakdown of the wood and a reduction in service life of man-made structures such as harbor pilings and boats^3^. The growth, reproduction and distribution of different types of shipworms varies, and their ecology and physiology are well studied^4,5^.

Microbial degradation of wood in marine waters has received less attention than that of marine borers because the degradation processes are much slower. But in environments where bivalves are physiologically excluded, these specialized fungi and bacteria become the key degraders^6^. Such environments include fresh waters, brackish waters, and anoxic waters^7–9^. Here wood can survive on the seabed for centuries and unique historic shipwreck can be recovered by marine archaeologists.

A large majority of marine wood degrading fungi are filamentous and belong to the group of Ascomycetes and Fungi imperfecti. During enzymatic breakdown of mainly cellulose and hemicellulose they form typical soft rot decay^1,10,11^. Marine wood degrading bacteria have been divided into two groups based on their morphological attack on the wood cell wall and are referred to as tunneling bacteria and erosion bacteria respectively^8,12^. A review on microbial degradation of wood in marine environments is given by Björdal (2012)^6^.

The oxygenated, very cold oceanic seas surrounding Antarctica are poor in wood material. As the prehistoric forest disappeared in the late Neogene^13^, the land has been without living trees and wood waste material in rivers and coast systems for 1702.5 Ma (million years). The quantities of driftwood from other continents coming into the system are very low, most likely due to the distance and the fact that circumpolar currents and Antarctic polar front represent a barrier for drift logs^14^. For this reason wood falls in Antarctic seas are extremely rare. Thus it is very interesting to ask whether wood degrading organisms are present and active in the coastal waters of Antarctic given the extreme rarity of their wood substrate.

Only a few studies have focusing on wood degradation in the Antarctic waters. One was carried out in 1980 with the aim of investigating the distribution of micro-fungi related to wood decomposition in the South Atlantic waters. Around 100 wooden blocks were submerged in deep 0.5 °C water for about 3 months near Signy Island. When the blocks were retrieved, wood inhabiting (lignicolous) marine fungi were isolated in lab and cultivated. The number of fungal isolation per block were few; 1,4 to 1,8 and two species could be identified as main colonizers^15^. This study provided the first indications of wood inhabiting fungi in the waters of Antarctic. The wooden blocks were not further examined for active degradation of the wood tissue, thus it is unknown if active wood degradation took place. As lignicolous fungi are defined as colonizers of the wood surface, they are not necessarily lignocellulolytic fungi (true wood degraders) able to decompose the wood components, lignin, cellulose and hemicellulose.

In 2009, an experiment was carried out in the waters of West Antarctic peninsula continental shelf. With help of advanced landers, wood and bone samples were deployed at two different depths for one year in order study biodegradation. Surprisingly no activity by wood borers was found, and there was no evidence of microbial decay. The wood was in pristine condition^14^. In another study, two pieces of wood were recovered from the sea floor, at about 20–30 m depth, at McMurdo Station, Antarctica. The oldest sample was from a wooden gangplank that fell off a ship and sank prior to 1960, and the biological colonization has been studied since 1967^16^. The second piece of wood was a section of new wood used to support a settling experiment that was placed in 1974. Pieces of each were retrieved in 2010 when it was noted that the latter piece still smelled fresh^17^. The authors found that none of the two wooden boards showed sign of “wood boring macrofaunal decomposers of any sort, nor any obvious microbial activity”. All studies so far show that biodeterioration of wood seems to be absent in the waters of Antarctic.

As microbial degradation can be hard to detect, especially if the decay is at its initial stages, this complementary study was undertaken to reexamine the two wooden boards from McMurdo Station^17^. This material had been conserved in alcohol that preserved the decay that eventually took place in the Antarctic waters as well as preventing new infections, which make it a perfect material for further studies. Our aim is to resolve if specialized wood degrading fungi and bacteria are active in the Antarctic waters.

## Results

### Wood species

The two samples were both identified as Douglas Fir (*Pseudotsuga*), a softwood species with typical helical structures within the cell walls^18^. Sample BI1332 had narrow annual rings compared to sample BI1333 with wide annual rings. Both appeared very strong and non-degraded apart from the softer outermost surface layer that also were discolored. Sample BI1332 was very dark brown whereas sample BI1333 appeared red-brown with clear iron contamination.

### Light microscopy

**Figure 1:**
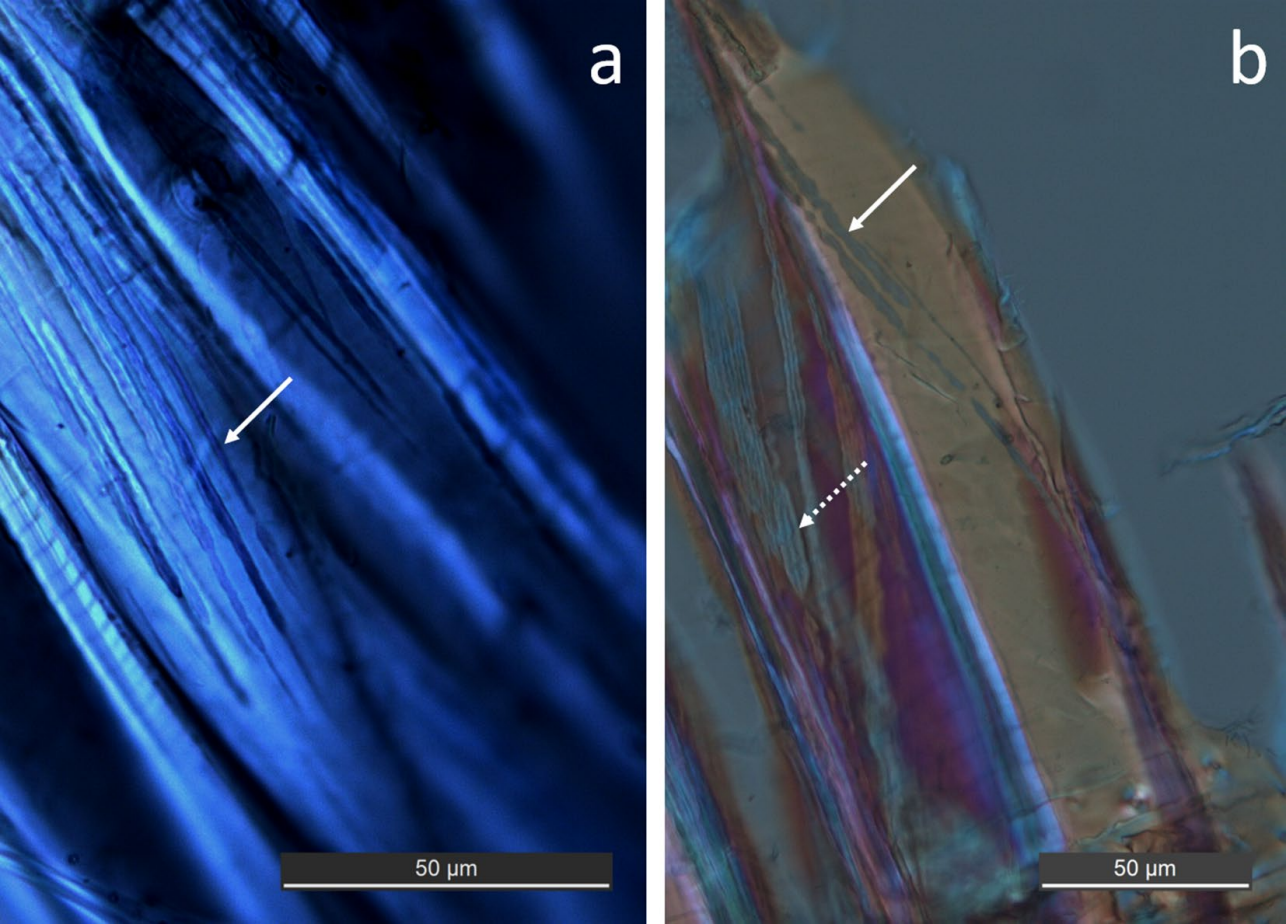
Typical soft rot cavities observed in fibres of samples BI 1333 (**a**) and samples BI 1332 (**b**). The hyphen are aligned along the microfibrils in the cell wall forming elongated thin cavities with pointed ends (arrows). The hyphen are present within the cavity (dotted arrow, **b**). Longitudinal sections observed with help of polarised light.

**Figure 2:**
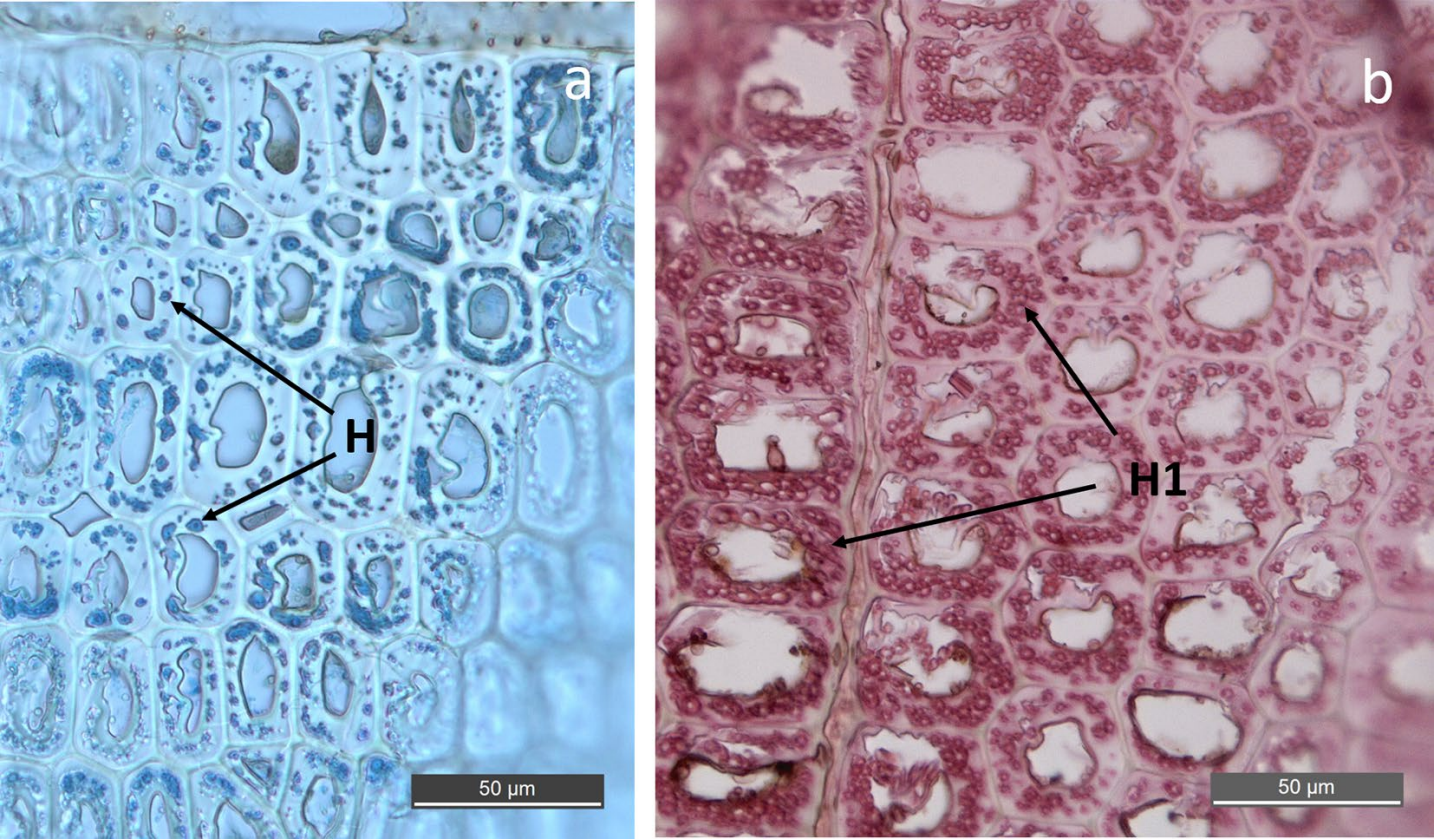
Micrograph shows typical SR decay observed in cross sections of BI 1332 (**a**) and BI 1333 (**b**). Characteristic irregular holes are located within the secondary cell wall. Each hole is a cross section of one hyphen (H). All fibers are heavily degraded and the most degraded cell walls contain a very high number thin narrow hyphen closely aligned which merged into clusters (H1).

Both wood samples showed decay caused mainly by soft rot fungi attack (SR) (Figs. 1, 2). The decay was concentrated to the outermost fibers and penetrated the first 2–3 mm of sample BI1332, and 2–4 mm of sample BI1333. Further in, the fibers were sound and non-degraded (Supplementary Fig. 1). Typical SR-cavities, following the micro-fibrillar orientation of the fiber, were observed in the cell wall of longitudinal sections (Fig. 1a, b). The hyphen linked to the formation of the cavity was also present indicating recent or ongoing degradation processes. Hyphen and cavities were extremely thin and closely aligned which also was evident from cross sections (Fig. 2a, b). The SR decay features observed in both boards were seemingly identical. Dark incrustations were observed in the outermost fibers of both samples (Supplementary material Fig. 2).

Both samples had indications of bacterial decay, type tunneling bacteria (TB) (Supplementary Material Fig. 3). Only smaller areas of TB attack were observed and a few were present next to SR cavities.

### SEM analyses

**Figure 3:**
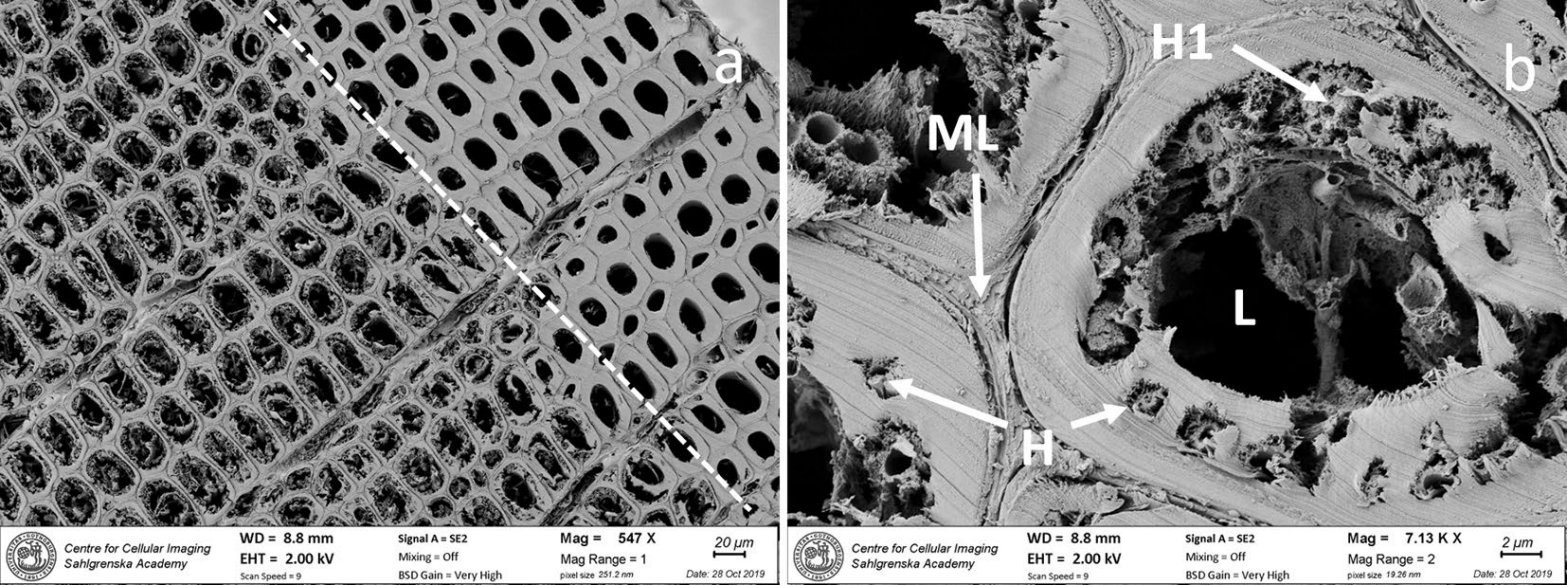
SEM micrograph shows extensive soft rot decay in the secondary cell wall. In dry state the disintegration and loss of cell wall material becomes more apparent. The forefront of decay (dotted line) meets the non-degraded area where fibres have sound cell walls (**a**). Close up shows individual hyphen surrounded by a narrow zone of enzymatic break down (**b**). As decay proceeds, the number of hyphen increases and merge (H) into clusters (H1). Some hyphen also grow in the lumen (L), whereas the middle lamella (ML) appears intact. Cross section of sample BI1333.

**Figure 4:**
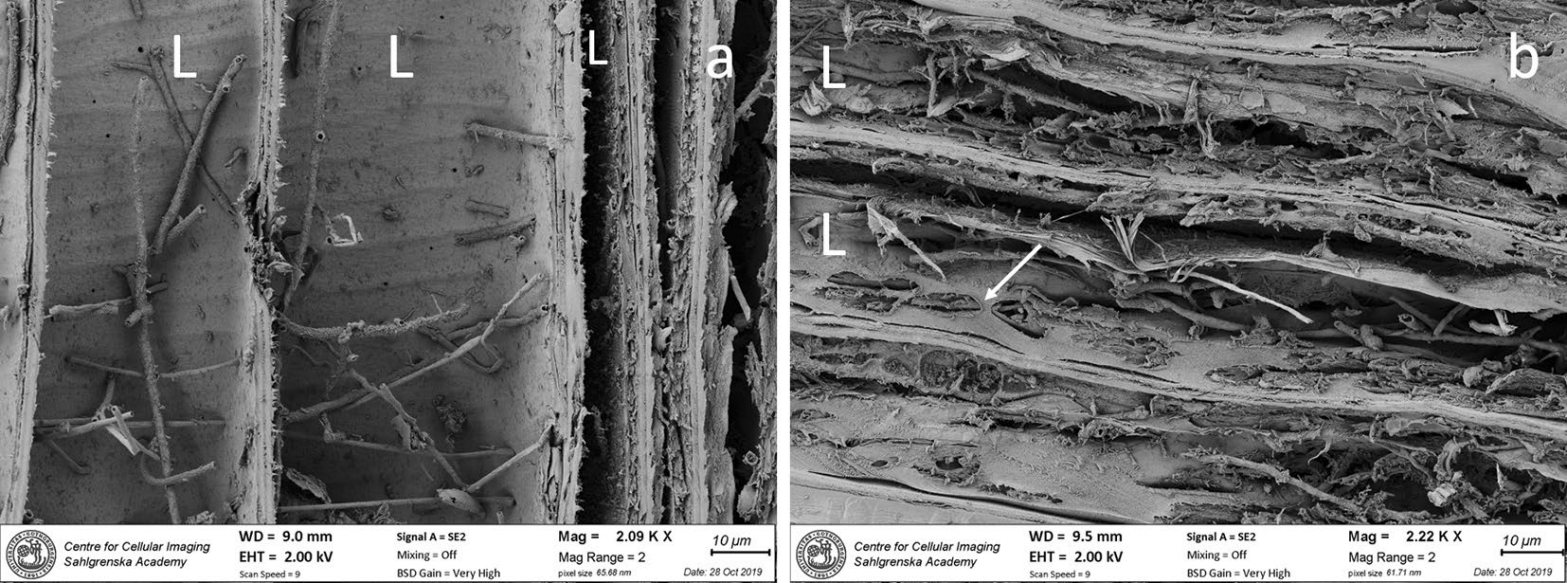
SEM micrograph shows growth and penetration of SR hyphen within and between the fibres Hyphal growth takes place in the lumen (L). Here they divide and spread into the secondary cell wall and into adjacent fibres by penetration (**a**). Within the cell wall the hyphen orientates themselves along the direction of the microfibrils and starts to decompose the wood. Cavities with pointed ends are formed during decay (arrow) (**b**)—a decay feature more easily distinguished with LM-technique (Fig. 1a, b). Longitudinal section of sample BI1333 (**a**) and BI1332 (**b**).

**Figure 5:**
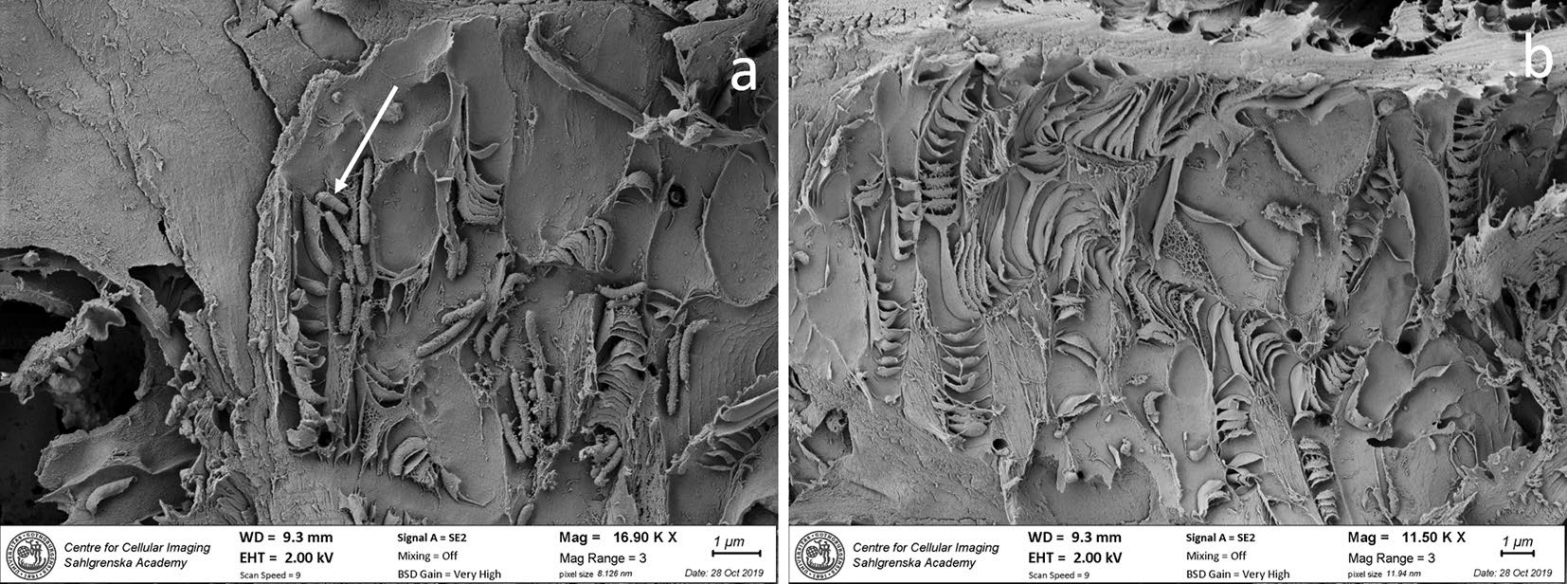
SEM micrograph shows degradation of cell wall by tunnelling bacteria. Within the cell wall, characteristic tunnels with closed chambers are formed during decay. Bacteria are closely attached to the wood matrix and encapsulated in chambers (arrow) (**a**). An advanced network of narrow tunnels of varying sizes, shapes and directions (**b**). Some chambers are extremely narrow. Longitudinal sections of sample BI13331.

SEM analyses did confirm the LM observations. SR decay were verified in both cross sections (Fig. 3a, b) and longitudinal sections (Fig. 4a, b). The diameter of the hyphen varied between 1 and 2 µm. In sample BI1333, minor bacterial cell wall degradation was verified. Here, irregular tunnels with narrow individual chambers and cross walls were formed by 0.5–1 µm long rod shaped bacteria (Fig. 5a, b). These decay features are typical for TB. In heavily degraded fibers from the outermost regions, secondary bacteria were present in voids and in residual material left behind by the primary degraders (SR and TB). Some were rod shaped and between 0.5 and 4 µm in length with varying thickness; others had spiral, cocci or “twisted” shapes (Supplementary material, Fig. 4a, b).

### Decay rates

As the light microscopy observation revealed a penetration of 2–3 mm of sample BI1332 after 50 years of exposure, and 2–4 mm of sample BI1333 after 36 years exposure, the decay rates are respectively 0.05, 0.08 mm/year. The same values correspond to respectively 2.5, 4.0 mm/ for 50 years. Both values are very low and could be regarded within the same interval as some natural variation could be expected.

## Discussion

It has been speculated that wood material does not degrade in the extreme cold waters of Antarctic^14,17^. However, we show that this assumption is not correct as we found active specialized lignocellulolytic fungi and bacteria degrading the wood cell walls of long-term submerged material. Further microbial activities in the wood was prevented as the samples were stored 50% alcohol after they were collected in 2010.

The anthropogenic wood samples of Douglas fir retrieved at two different sites outside McMurdo station showed ongoing microbial decay in both samples after 50 and 36 years of exposure at approximately 20 m depth. The two samples showed similar decay profiles dominated by an extensive decay by soft rot fungi in the outermost 2–4 mm of the board (Figs. 1, 2, 3, 4) accompanied by discrete attack of tunneling bacteria (Figs. 5, Suppl Fig. 3). No other types of wood decay were observed. The SR hyphen could be correlated to individual SR cavities (Figs. 1a, b, 4b) and TB were found in their characteristic burrows (Fig. 5a, b). Both features are indicative of ongoing degradation.

The history of the two wooden boards were different and it is not known whether the gangplank could have been infected and degraded during its presumable long time on board a ship. In contrast, the construction board was new and sound when it was used in an underwater/seabed construction; therefore it was not degraded at the start of exposure. When our analyses showed that both boards were degraded to an almost equal extend and had the same decay profile it strongly suggest that that gangplank did not have any “pre-infection” of importance. This conclusion is supported also by the fact that both boards were made by the same wood species (Douglas fir), exposed in the same waters at a similar depth, and for a comparable amount of time. We therefore conclude that decay observed in both boards is a consequence of marine seabed exposure in the water of McMurdo.

The SR decay pattern was very distinctive (Figs. 1, 2, 3, 4) and matched with morphological features typical of SR, type I, found in both marine and terrestrial environments^12^. The SR type I is characterized by an initial hyphal infestation in the lumen followed by penetration and growth within the secondary cell wall that is rich in holocellulose. Hyphen follows the orientation of the microfibrils, which results in characteristic helical elongated SR cavities formed during decay^19–21^. Marine SR decay are generally often concentrated in the outermost layers of the surface, as observed in baits exposed to diverse seas in an international study^22^, and shipwreck timbers^23,24^. The wood surface will soften and often darker^6^.

Two features might stand out for Antarctic SR decay. These are (1) the decay rate, and to a lesser extend (2) the thickness of the hyphen and cavities produced by the fungi. Compared to decay rates of wood exposed in other marine waters, a penetration of 2–3 mm in 35-, respectively 50 years represents extremely slow activity. Comparable data from saline marine trials at the British coast shows between 1 and 2.4 mm penetration of SR fungi after only 40 weeks of exposure (about 3 mm/year) and a weight loss about 30% in pine samples^25^. This is supported by laboratory studies running for 24 weeks where up to 25.9% dry mass loss was evident after decay test of 14 marine SR isolates^26^. This means that annual decay rate about 0.05 mm/year at McMurdo compared to 3.0 mm/year at the British Coast is almost two orders of magnitude slower in the Antarctic. This correlates with the unpublished slow bacterial growth rates observed in McMurdo Sound at 43 m in 1989 (J. Hansen & P. Dayton, personal communication). The second feature; the very thin and closely aligned SR cavities (Figs. 2, 3), could be a response to adaptation in an extreme environment. Morphological change and de-sizing of microorganisms have been described in energy poor environments were “maintenance state” and “survival state” are suggested ^27^.

TB are cosmopolitans and ultimate wood degraders, as they do degrade preservative treated wood, as well as very durable timbers in aquatic as well as in terrestrial environments^20,28^. The identity of these rod-shaped bacteria are still not known. The morphological decay features of the chamber forming TB (Fig. 5a, b) were in principle identical to those found in earlier SEM (scanning electron microscopy) studies on TB^23,29–32^. However, the chambers appeared extremely narrow, which might indicate a more repressed growth in the Antarctic waters. Further details on the TB decay process on a cellular level are given in TEM (transmission electron microscopy) studies^20,33^.

We found TB degradation taking place close to SR decay and occasionally within the same fiber. This is a common observation in marine trials, but not often reported. A long term experiment at the west coast of Sweden showed that both SR and TB were simultaneous degrading wood (pine, oak and birch) after only 6 and 12 months in the water column^34^. Decay decreased 10 cm below seabed and only erosion bacteria were found able to degrade wood in the anoxic sediment layers at 48 cm depth. This emphasizes the need of dissolved oxygen for growth by SR and TB; something that was available in the waters of Antarctic, where concentration of oxygen are close to saturation reflecting the annual phytoplankton blooms^35^.

Inside the wood material, at the boundary zone where degraded and still non-degraded tissue meet (Fig. 3a), initial decay by SR fungi was the only evidence of microbial life. In contrast, a large numbers of secondary bacteria and/or scavengers were present in the heavily degraded fibers from the surface area (Supplementary Fig. 4 a, b). Some had spectacular forms, like the twisted short rods and the screw/spiral-formed. The latter have also been observed in submerged timbers from the shipwreck Defence on the east coast of US.^36^. These observations confirm that the microbial community associated with wood degradation increases in size and diversity as decay by primary degraders (SR, TB) proceeds^8,33^. Recent complementary and in-depth information from DNA analyses on wood falls at varying depths reports on complicated ecosystems and significant bacterial diversity and succession within the wood surface after relatively short time of immersion ^37–40^. Similar type of data would be most interesting from the Antarctic region.

The global spread of marine fungi is mainly related to the temperature and salinity of the water as well as the depth. Some temperate- and tropical seas contain many lignicolous species whereas the Antarctic seas have very few species. This could related both to the extreme environment as well as to the fact that this area has been less explored by scientists^41^. In 2000, a large number of lignicolous marine fungi were tested in laboratory experiments for evidence of active wood decay. Out of 84 species, a total of 30 did form soft rot decay (Ascomycota, Mitosporic (Fungi Imperfecti), and 2 species white rot decay (Basidiomycota)^11^. The still most comprehensive list, containing 59 marine soft rot producing fungi are given by Mouzouras in 1989, although some species have been added during the last 40 years and more await identification^42^. The Soft rot fungus degrading the wood from Antarctic may represent novel species. No previous soft rot decay has been reported from Antarctic waters, but there is evidence of a few lignicolous fungi. In 1985, lignicolous fungi was isolated from the waters of sub-Antarctic Signy Island and two fungi were dominant; *Monodictys pelagica* (T.W. Johnson) E.B.G. Jones, 1963) and *Ceriosporopsis tubulifera* ((Kohlm.) P.W. Kirk ex Kohlm., 1972) ^15^. Both species are today listed as typical marine soft rot degraders in a variety of waters ^11,21^. *M.pelagica* have a wide salinity tolerance ^43^ and an extensive distribution in oceans and described among the most common fungi imperfecti in the northern colder waters ^44^, although it also is observed from the southern hemisphere at the cold waters of Chile ^45^. *M. pelagica* has also been identified as serious degraders of timbers from the historic warship Mary Rose, UK as well as potential degrader of shipwrecks in the Baltic Sea^6^. *Ceriosporopsis tubulifera* have been reported from the Arctic region^46^.

Our examination of McMurdo wood samples has demonstrated activity and growth of lignocellulolytic soft rot fungi and tunneling bacteria in the cold Antarctic waters. For wood material and wooden shipwrecks situated in these waters the absence of marine borers prolong the lifespan of this organic material. But as shown, a slow microbial deterioration takes place and will continue over time until wood is totally decomposed. This situation is in many aspects similar to the degradation process observed in the brackish Baltic Sea, where many unique historic shipwrecks are found seemingly well preserved by marine archaeologists^47^. Here, analyses have shown decay by both SR, TB, and erosion bacteria and a slow transformation of solid wood takes place, leading to a continuous breakdown of the surface layers^6,24,48,49^. For the historic ships including Nordenskjold’s Antarctic that sank in 1902 and Shackleton’s *Endurance* that sank in the waters of Antarctic in 1915, decay processes might be slightly slower, as the rate observed are around 3 mm/ 50 years, compared to soft wood from the Vasa wood that are more than 20 mm/300 years (3.3 mm/50 years). But decay rates are complicated because they are found to be dependent on several factors, some related to the wood itself, others to environment, and finally to the time of exposure^6^.

## Conclusion

The wood retrieved outside McMurdo station showed active microbial degradation after 36 and 50 years of exposure in the cold waters of Antarctic. The two samples showed similar degradation profiles dominated by an extensive decay by soft rot fungi in the outermost 2–4 mm of the board accompanied by minor decay by tunneling bacteria. The soft rot hyphen could be correlated to individual soft rot cavities and tunneling bacterial were found in their characteristic burrows. Both features observed by light microscopy and scanning electron microscopy are indicative of ongoing degradation. No other types of wood decay were observed. This study shows for the first time that although marine borers seems absent from these extreme waters, specialized lignocellulolytic marine microorganisms are highly active.

## Material and methods

### Material

Two wooden boards from the marine waters close to McMurdo station at the Antarctic were available for analyses. Both were stored in 50% ethanol since their recovery in 2010 in order to avoid contamination, preserve the samples, and exclude further activity of microorganisms. One subsample was sawn from the gangplank BI 1332 (Hut Point, submerged in marine water for over 50 years) and another from the construction board BI 1333 (McMurdo submerged for 36 years) and sent from the Scripps Institution of Oceanography submerged in 50% ethanol. We added 50% ethanol and stored at 8 °C on arrival. The thickness of board BI1332 and BI1333 were 14 mm, 40 mm respectively. From the sawn end of each subsample, one piece was taken for investigation (approx. 40 × 20 × 10mm). Both pieces did include the original wood surface.

The gangplank was dropped around 1960 by a U.S Navy fuel tanker that was probably manufactured before or during WW II. Wooden gangplanks can be transferred from ship to ship or replaced with new ones, so the actual age was not possible to determine. It appeared very worn but not degraded in 1967 when first inspected and it appeared the same condition in 2010. It could have been subject to a great deal of ocean spray and may have fallen into the sea before it was submerged at McMurdo Station. The other piece of wood was transported to McMurdo Station in 1974 to be used in construction. It was fresh and new on arrival and had never been near the ocean until it was deliberately imbedded in the seafloor, September, 1974. Both wooden boards are further described and illustrated in Dayton et al.^17^ and in Dayton et al.^16^.

### Methods

#### Light microscopy

From each piece, small and very thin sections were taken by hand with double edged razorblade in all three directions (transversal, tangential and radial), and did include sections from the outermost surface layers as well as tissue from the interior parts. The thin sections were stained with either: 0.1% Aniline blue in 50% lactic acid; 0.1% Astra Blau in 50% lactic acid; or 0.1% Safranin in glycerol. Observations were carried out by light microscopy at magnifications up to × 630 and with frequent use of polarized light.

#### SEM (scanning electron microscopy)

Prior to SEM analyses, small blocks up to 3 mm in length and 2 mm in height was taken by hand with double edged razor blade in both transversal and radial directions. This was followed by fixation, dehydration, mounted on stubs, and gold coated using *routine protocol*^50^. SEM instrument used for observations was Zeiss Gemini 450.

## Supplementary information

Supplementary Legends

Supplementary Figure 1.

Supplementary Figure 2.

Supplementary Figure 3.

Supplementary Figure 4.
