# Supplementary material for: First evidence of microbial wood degradation in the coastal waters of the Antarctic
Source: Sci Rep. 2020 Jul 29;10:12774. doi: 10.1038/s41598-020-68613-y (PMC7391713; doi:10.1038/s41598-020-68613-y)
Supplement: Supplementary file 1 — Supplementary Legends [file 41598_2020_68613_MOESM1_ESM.docx]

**First evidence of microbial wood degradation in the coastal waters of the Antarctic**

Charlotte G. Björdal ^1*^; Paul K. Dayton ^2^

**Legends supplementary figures 1 – 4**

Supplementary figure 1

LM micrograph shows non-degraded tissue observed 4mm from the wood surface. The thicker cell wall of the latewood (LW) fibers are still not attacked although thin network of hyphen (arrow) are invading the lumen of earlywood (EW) fibers in the upper left corner. Characteristic resin canals (R) are observed among the latewood fibers. Cross section of sample BI1332.

Supplementary figure 2

Dark incrustation, most likely iron related compounds, discolour the section and are accumulated especially in rays (R) and in structural elements, like the spiral thickenings (S) in the cell lumen. Longitudinal tangential section of sample BI1333 observed by LM.

Supplementary figure 3

LM micrograph shows extensive spread of non-oriented minute tunnels (arrows) disintegrating the cell wall matrix. The decay pattern indicate activity by tunnelling bacteria. Longitudinal section sample BI1332.

Supplementary figures 4 a,b

SEM micrographs shows secondary bacteria situated in residual material in heavily degraded fibres from the surface layer of sample BI1332. A variety of morphological form and sizes are evident such as slim and thick rods with normal, slightly twisted, or spiral curves. Granular features appears on the body of ca 1µm large rods (fig. 4 a).
