# Supplementary figures and images for: First evidence of microbial wood degradation in the coastal waters of the Antarctic
Source: Sci Rep. 2020 Jul 29;10:12774. doi: 10.1038/s41598-020-68613-y (PMC7391713; doi:10.1038/s41598-020-68613-y)

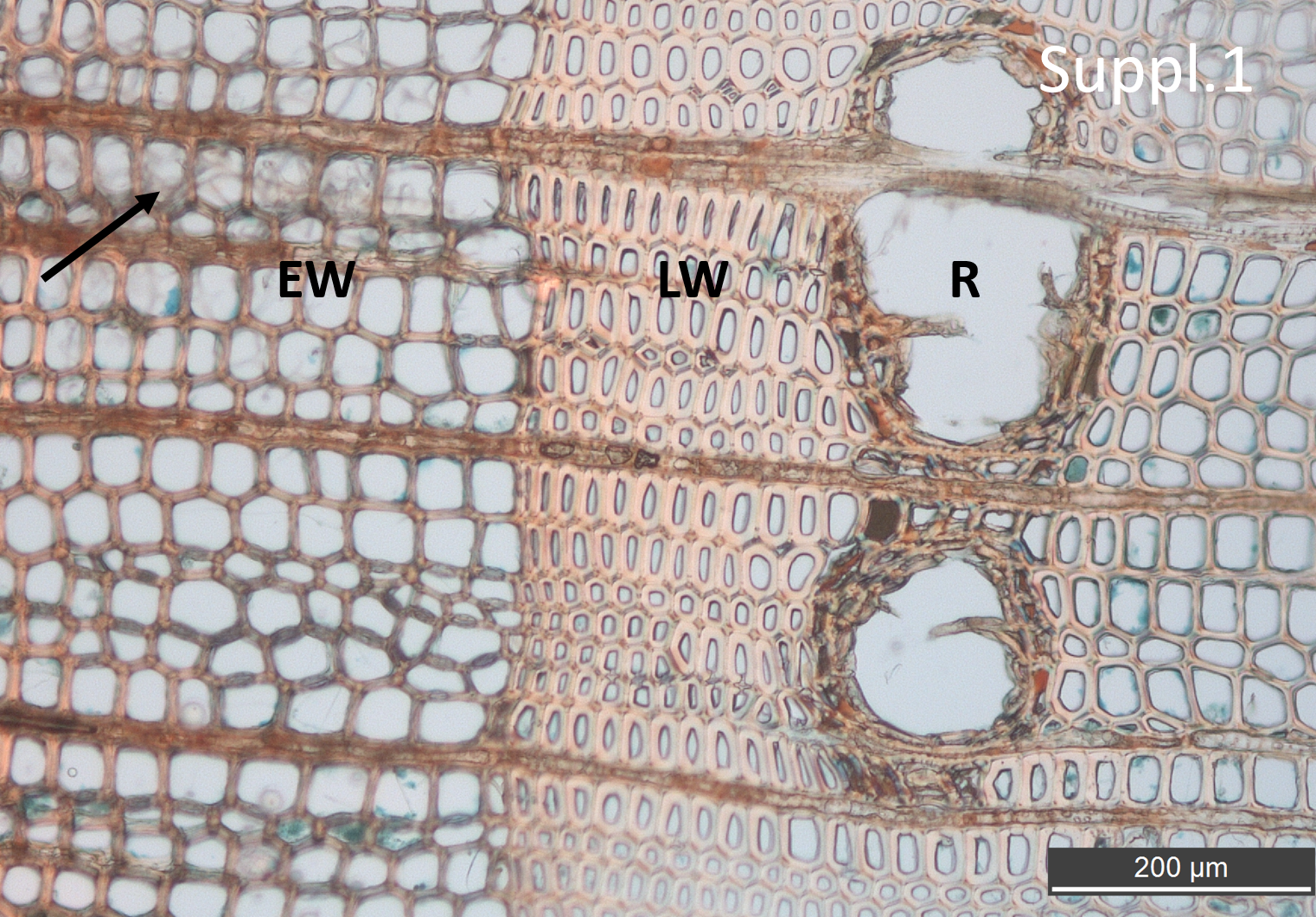

Supplement: Supplementary file 2 — Supplementary Figure 1. [file 41598_2020_68613_MOESM2_ESM.tif]

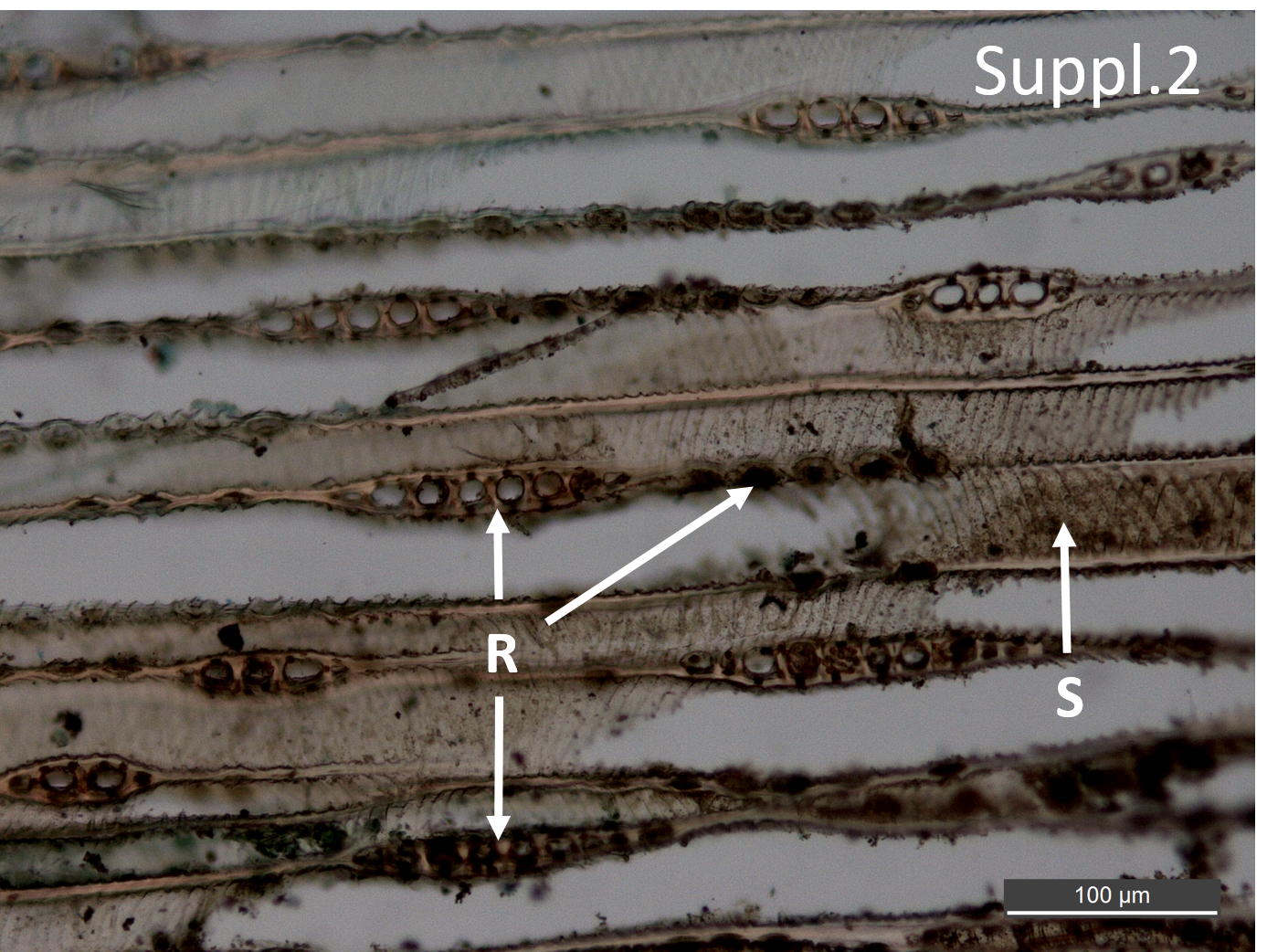

Supplement: Supplementary file 3 — Supplementary Figure 2. [file 41598_2020_68613_MOESM3_ESM.tif]

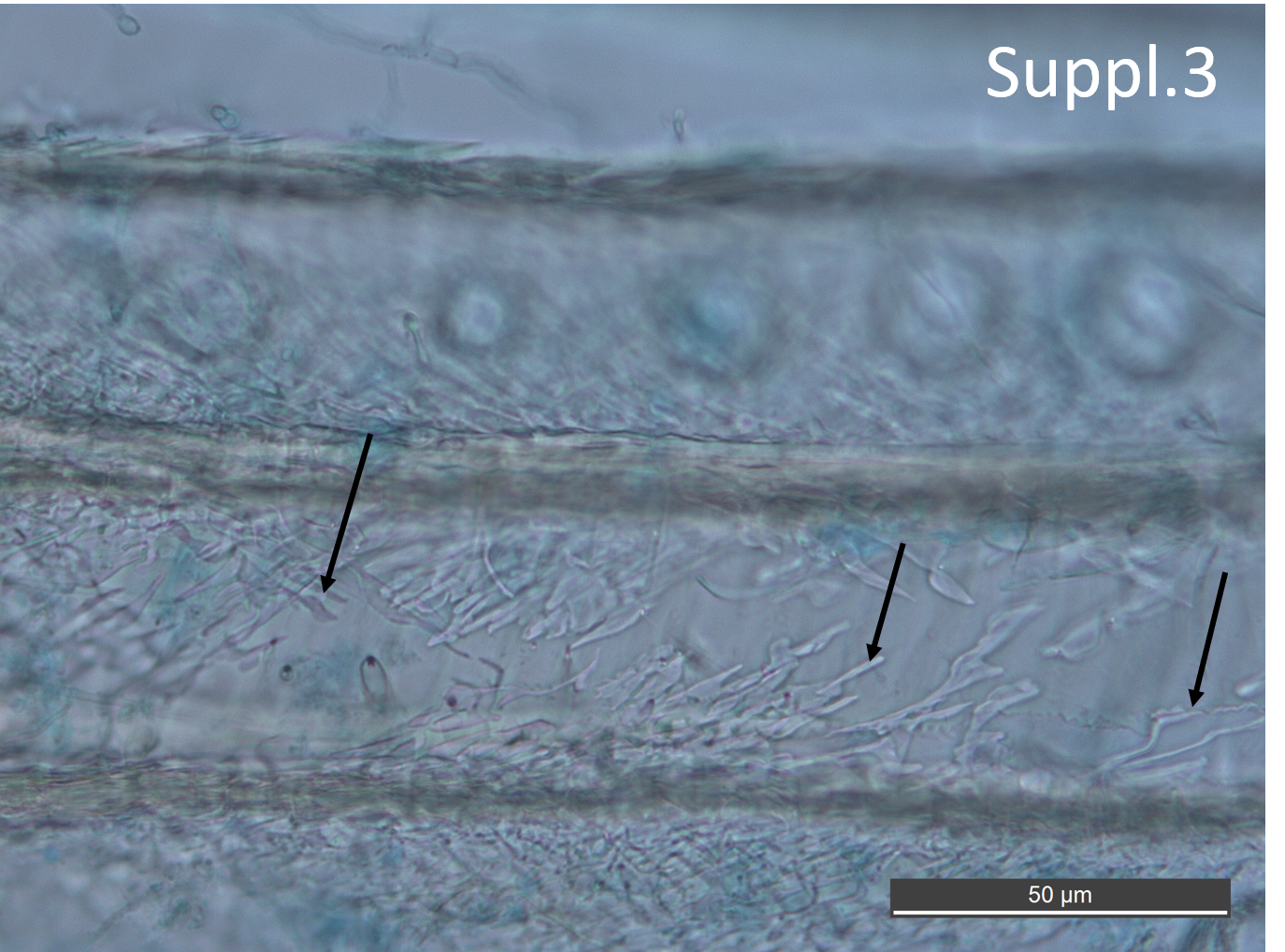

Supplement: Supplementary file 4 — Supplementary Figure 3. [file 41598_2020_68613_MOESM4_ESM.tif]

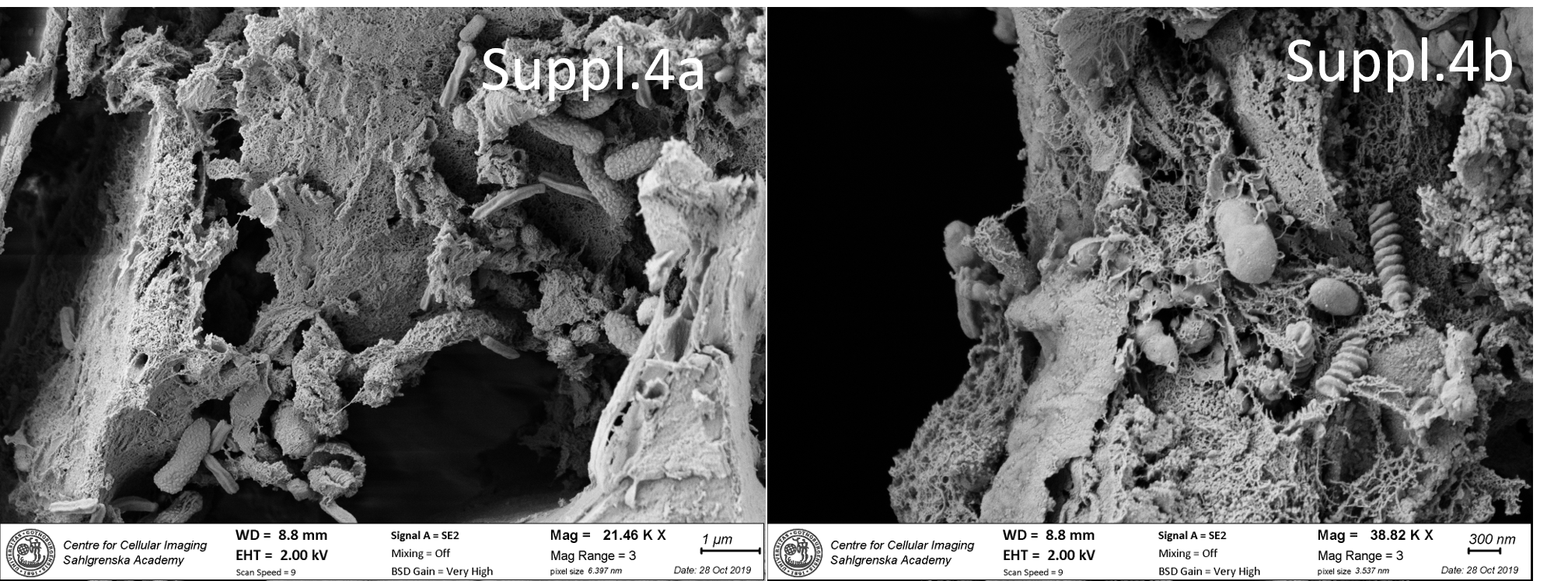

Supplement: Supplementary file 5 — Supplementary Figure 4. [file 41598_2020_68613_MOESM5_ESM.tif]
